# Evaluation of Jute–Glass Ratio Effects on the Mechanical, Thermal, and Morphological Properties of PP Hybrid Composites for Sustainable Automotive Applications

**DOI:** 10.3390/polym17243335

**Published:** 2025-12-17

**Authors:** Tunahan Özyer, Emre Demirci

**Affiliations:** 1 Department of Mechanical Engineering, Bursa Technical University, Bursa 16310, Türkiye; tunahan.ozyer@tofas.com.tr; 2 R&D Center, TOFAS Turkish Automobile Factory, Bursa 16110, Türkiye; 3 Automotive Application and Research Center, Bursa Technical University, Bursa 16310, Türkiye

**Keywords:** polypropylene composites, hybrid biocomposites, jute–glass fibers, sustainable materials, characterization, automotive applications

## Abstract

This study investigates polypropylene (PP)–based biocomposites reinforced with systematically varied jute and glass fiber ratios as sustainable, lightweight alternatives for semi-structural automotive parts. Four formulations (J20/G0, J15/G5, J10/G10, J5/G15) with a constant 20 wt% total fiber were produced by injection molding and characterized through mechanical, thermal, and morphological analyses. Tensile, flexural, and Charpy impact tests showed progressive improvements in strength, stiffness, and energy absorption with increasing glass fiber content, while ductility was maintained or slightly enhanced. SEM revealed a transition from fiber pull-out in jute-rich systems to fiber rupture and stronger matrix adhesion in glass-rich hybrids. Thermal analyses confirmed the benefits of hybridization: heat deflection temperature increased from 75 °C (J20/G0) to 103 °C (J5/G15), and thermogravimetry indicated improved stability and higher char residue. DSC showed negligible changes in crystallization and melting, confirming that fiber partitioning does not significantly affect PP crystallinity. Benchmarking demonstrated mechanical and thermal performance comparable to acrylonitrile–butadiene–styrene (ABS) and acrylonitrile–styrene–acrylate (ASA), widely used in automotive components. Finally, successful molding of a prototype exterior mirror cap from J20/G0 validated industrial processability. These findings highlight jute–glass hybrid PP composites as promising, sustainable alternatives to conventional engineering plastics for automotive engineering applications.

## 1. Introduction

The automotive industry, as one of the most innovation-driven sectors, is increasingly focused on reducing environmental impact, improving fuel efficiency, and ensuring recyclability in vehicle design. These targets have accelerated the shift away from metals and conventional plastics toward lighter and more sustainable alternatives. Polymer composites have emerged as promising candidates due to their favorable strength-to-weight ratio, chemical resistance, and design flexibility, which enable weight reductions of up to 65% and associated CO_2_ savings [1,2,3,4,5,6]. In parallel, natural fiber–reinforced composites provide additional advantages such as biodegradability, renewability, and low embodied energy, making them particularly attractive within global sustainability frameworks [7,8,9]. Furthermore, studies on crashworthiness have demonstrated that jute- and glass-fiber composites can achieve competitive specific energy absorption and crush force efficiency, confirming their potential for structural applications [10]. Beyond mechanical performance, lifecycle assessments highlight that polymer composites significantly reduce emissions and manufacturing energy requirements, reinforcing their role in environmentally responsible automotive production [11,12,13,14]. Collectively, these findings indicate that polymer composites not only satisfy performance standards but also provide viable pathways toward sustainable mobility [15,16].

Given these advancements, Polypropylene (PP) has emerged as one of the most widely used thermoplastic matrix materials in the development of sustainable and high-performance automotive components. Its widespread adoption is attributed to key advantages such as low density, cost-effectiveness, ease of processing, and chemical resistance, which make it especially attractive for applications in the automotive, packaging, and construction sectors [17,18]. However, neat PP has inherent limitations in terms of mechanical strength, stiffness, and thermal stability, which restrict its standalone use in demanding structural applications [19]. In addition, PP possesses inherently low surface free energy, which complicates painting, coating, and adhesive bonding; therefore, surface treatments such as corona, plasma, or primers are typically required in automotive applications. While this surface-energy limitation is usually addressed through such post-processing steps, the mechanical and thermal deficiencies can be effectively mitigated by reinforcing PP with natural or synthetic fibers, resulting in composites with enhanced modulus, strength, and thermal resistance. For instance, studies have shown that incorporating natural fibers such as jute, hemp, or bamboo into PP matrices not only improves mechanical and thermal performance but also contributes to better environmental sustainability [20,21]. Therefore, PP remains a highly promising matrix material in the development of lightweight, cost-effective, and semi-structural composite components, especially when optimized through appropriate fiber reinforcement and compatibilization strategies.

To overcome the limitations of neat PP and natural fiber–reinforced composites, recent studies have explored hybrid systems that combine natural and synthetic fibers within PP matrices to balance mechanical performance with sustainability. Natural fibers such as jute, flax, hemp, and sisal offer advantages including low density, renewability, and biodegradability, but are hindered by hydrophilicity, poor interfacial adhesion with PP, and variability in mechanical properties [22,23,24]. Synthetic fibers, particularly glass fibers, provide superior strength and thermal stability, making them suitable for higher-performance demands. Hybridization leverages the complementary benefits of both fiber types, with synthetic fibers contributing stiffness and thermal resistance while natural fibers reduce cost and environmental footprint [25,26,27].

Among these systems, jute–glass fiber–reinforced PP composites have received increasing attention. Reported studies indicate marked improvements in tensile strength, flexural modulus, and impact resistance compared to natural fiber–only systems, with flexural modulus enhancements of up to 130% over neat PP and water absorption below 0.3% [28]. Optimized jute–glass hybrids can even achieve properties comparable to jute–carbon systems, offering a more sustainable and cost-effective solution [29]. Additionally, fiber orientation and layering play a key role: placing glass fibers in outer layers enhances flexural and tensile performance, while jute fibers improve impact resistance and maintain sustainability benefits [30]. Injection-molded hybrids using long jute and short glass fibers have also shown significant toughness and stiffness gains, though fiber distribution remains critical [31]. Overall, jute–glass PP hybrids enable tailored property profiles that align with the structural and sustainability requirements of modern automotive engineering [32].

The ratio of natural to synthetic fibers in hybrid composites is a decisive factor governing both mechanical and thermal behavior. Increasing the fraction of high-modulus synthetic fibers, such as glass or carbon, typically enhances tensile and flexural properties as well as thermal stability, owing to their higher load-bearing capacity and resistance to heat [33,34]. In contrast, higher natural fiber contents reduce density, cost, and environmental footprint but may compromise stiffness and thermal resistance while increasing moisture uptake due to their hydrophilicity [22,35]. Several studies highlight that balanced hybrid configurations achieve the most favorable trade-off, where synthetic fibers contribute stiffness and thermal stability, while natural fibers enhance impact resistance, weight reduction, and sustainability [36,37]. This balance is particularly evident in hybrid systems where moderate glass fiber contents enhance tensile and flexural moduli by over 20%, while maintaining adequate toughness and limiting water absorption to acceptable levels for semi-structural applications.

Several studies have explored jute–glass fiber hybrid composites with different matrices, fiber ratios, fabrication techniques, and testing protocols, providing diverse insights into their mechanical and thermal behavior. A considerable body of work has focused on PP–based hybrids. Sommer et al. [38] studied compression-molded PP composites with 30 wt% total fiber and reported that replacing glass with jute reduced density and enhanced sustainability, though tensile and flexural strengths decreased moderately. Khan et al. [39] compared woven hessian jute/PP and woven E-glass/PP composites (50 wt% fiber each), showing that glass/PP exhibited nearly twice the tensile strength and higher interfacial shear strength (IFSS) due to superior fiber–matrix adhesion. Wang et al. [40] investigated hydrothermal aging of injection-molded long-fiber PP composites with varying jute–glass ratios; increasing jute content raised tensile modulus but reduced tensile strength and markedly increased water uptake, while glass-rich systems retained strength under humid conditions. Uawongsuwan et al. [31] analyzed the effect of jute fiber size and shape in injection-molded long-fiber thermoplastic PP hybrids, all containing 10 wt% glass. Glass addition increased tensile modulus by 4–18% and flexural modulus by 16–30%, with re-pelletized jute fiber/polypropylene (JF/PP) showing the highest improvements (tensile +64%, flexural +74%, impact +948%), attributed to stronger glass fibers and better fiber orientation, though jute aggregation and poor alignment remained limiting factors. Khan et al. [41] developed short jute (2–3 mm) and short E-glass fiber PP composites (20 wt% fiber) via compression molding, reporting 32 MPa tensile and 38 MPa flexural strength for JF/PP, which degraded substantially after soil burial, unlike glass/PP, which retained most properties. Ravishankar et al. [42] fabricated PP composites with 40 wt% fiber, including pure jute (40:0), jute–glass (20:20), jute–carbon (20:20), and ternary jute–glass–carbon (20:10:10). Glass addition produced substantial mechanical gains, further enhanced by carbon, with the ternary hybrid approaching carbon-only performance. Complementing these, Kshatriya et al. [43] reviewed jute- vs. glass-reinforced PP, showing that glass/PP offers superior mechanical strength and stiffness, while JF/PP provides biodegradability and lower environmental impact. Despite these contributions, studies on jute–glass PP hybrids with comprehensive thermal analyses, particularly using both Thermogravimetric analysis (TGA) and Differential scanning calorimetry (DSC), remain scarce, whereas many investigations have instead focused on bamboo [44], banana [45], or kenaf [25] with glass fibers.

Beyond PP matrices, similar investigations on epoxy and polyester systems provide broader insights into jute–glass hybridization. In epoxy-based composites, increasing the glass fiber fraction enhances stiffness, tensile strength, and thermal stability, while higher jute content improves impact resistance and reduces density [46,47,48]. In polyester-based systems, hybrid laminates containing both jute and glass fibers exhibit similar trends: stacking sequence and fiber treatment significantly influence performance, with glass-rich layers improving strength and jute-rich configurations enhancing toughness and energy absorption [49,50,51]. Overall, these studies show consistent strength and toughness contributions of glass and jute fibers across polymer matrices, supporting the present hybrid design.

Many studies have examined hybrid composites combining glass with natural fibers such as bamboo, banana, and kenaf in matrices like epoxy or polyester, clarifying how hybridization influences mechanical strength, stiffness, and thermal stability. However, for PP composites reinforced with both jute and glass fibers, systematic thermal characterization remains limited. In particular, comprehensive analyses incorporating TGA, DSC, and heat deflection temperature (HDT) are scarce, and to the best of the authors’ knowledge, quantitative HDT data for jute–glass PP hybrids have not been reported in the literature. Addressing this gap, the present study provides an integrated evaluation of mechanical, thermal (TGA, DSC, HDT), and morphological (scanning electron microscopy (SEM)) properties across systematically varied jute/glass ratios.

Beyond filling this scientific gap, the study also considers industrial relevance. Acrylonitrile–butadiene–styrene (ABS) and Acrylonitrile–styrene–acrylate (ASA) are among the most widely used thermoplastics in the automotive industry due to their favorable balance of strength, impact resistance, and weatherability [52]. Yet, increasing environmental pressures and regulatory demands have intensified the need for more sustainable alternatives [53]. By benchmarking the performance of jute–glass PP hybrids against ABS and ASA, this work offers a distinct contribution to understanding their feasibility for semi-structural automotive applications. Collectively, the results underscore both the scientific significance and practical value of jute–glass PP hybrids as sustainable, high-performance materials.

## 2. Materials and Methods

### 2.1. Materials

The base polymer matrix was a commercial polypropylene copolymer grade, Tecolen PP CP30 (Eurotec Engineering Plastics, Tekirdağ, Türkiye), heat- and UV-stabilized for automotive applications, with a density of 0.90 g/cm^3^ and a melting temperature in the range of 160–165 °C. In the present study, all PP-based biocomposites were compounded by Eurotec Engineering Plastics (Tekirdağ, Türkiye) according to the target jute/glass fiber ratios defined in this work, using this copolymer matrix. To examine hybridization effects at a fixed overall reinforcement level, the total (jute + glass) fiber content was maintained at 20 wt%, while the glass-fiber fraction was systematically increased with a corresponding reduction in jute. Specimen identifiers followed the notation Jx/Gy, where x and y denote the jute and glass mass fractions (wt%) of the composite, respectively: J20/G0, J15/G5, J10/G10, and J5/G15; the nominal compositions are summarized in Table 1. Both the jute fibers and the short glass fibers (13 µm in diameter, chopped strands) used in these formulations were supplied by Eurotec Engineering Plastics (Tekirdağ, Türkiye) as part of the compounded materials.

**Figure 1:**
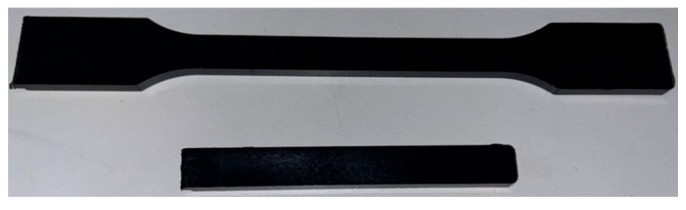
Geometries of the test specimens.

The biocomposites were processed on an industrial co-rotating twin-screw extruder (ZSK26 MC, Coperion GmbH, Stuttgart, Germany) with a screw diameter of 26 mm and an L/D ratio of 40. Compounding was carried out using a barrel temperature profile of 190–210 °C. Prior to injection molding, the compounded materials were dried at 80 °C for 4–8 h to minimize moisture-related variability. Test specimens were injection molded with the following processing parameters: feed-throat temperature < 60 °C, barrel temperature 190–210 °C, mold temperature 20–40 °C, and holding pressure 40–80 MPa. Tensile specimens conformed to ISO 527-2:2012 Type 1A [54], and rectangular ISO bars with nominal dimensions 80 × 10 × 4 mm were molded; the specimen geometries are shown in Figure 1. The densities of the composites were measured according to the ISO 1183-1 standard [55].

### 2.2. Mechanical Tests

#### 2.2.1. Tensile Test

Tensile tests were conducted on Type 1A specimens in accordance with ISO 527-1:2019 (general principles) [56] and ISO 527-2:2012 [54] (test conditions). Experiments were performed on a Shimadzu AGS-X universal testing machine (Kyoto, Japan) equipped with a 10 kN load cell. According to ISO 527-1 and ISO 527-2, the crosshead speed was set to 1 mm/min for determining the elastic (Young’s) modulus and then increased to 50 mm/min and maintained until fracture, consistent with practices reported in prior studies [57,58]. During testing, force–displacement data were recorded synchronously and converted to engineering stress–strain curves in Trapezium (version 1.3.1) software using the nominal cross-sectional area and gauge length. The elastic modulus was calculated by linear regression over the initial linear portion of the stress–strain curve, corresponding to a strain interval of approximately 0.05–0.25%, as specified by ISO 527-1. The ultimate tensile strength and elongation at break were determined from the characteristic points of each curve.

#### 2.2.2. Three-Point Flexural Test

Three-point flexural tests were performed in accordance with ISO 178:2019 [59] on rectangular specimens of 80 × 10 × 4 mm. Tests were conducted on a Shimadzu AGS-X universal testing machine (Kyoto, Japan) equipped with a 10 kN load cell. The crosshead speed was 2 mm/min, and the support span (L) was set to 64 mm. Specimens were placed on two lower supports with a span of 64 mm and loaded centrally at the mid-span while force–deflection data were recorded for analysis. The flexural strength ($\sigma_{f}$) and the flexural modulus ($E_{f}$) were evaluated according to Equations (1) and (2):

(1) $$\sigma_{f}=\frac{3F_{max}L}{2bh^{2}}$$

(2) $$E_{f}=\frac{L^{3}m}{4bh^{3}}$$

where is $F_{max}$ the maximum applied load, L the support span, b the specimen width, h the specimen thickness, and m the slope of the initial linear portion of the load–deflection curve.

#### 2.2.3. Charpy Impact Test

Impact resistance was evaluated in accordance with ISO 179-1:2023 [60] on Type 1 specimens tested in the edgewise, unnotched configuration (1 eU, 80 × 10 × 4 mm). Tests were carried out at room temperature on an Instron Ceast 9050 pendulum impact tester (Pianezza, Italy) equipped with a 5 J hammer. Specimens were supported in the Charpy fixture and struck at mid-span; the absorbed energy (J) was recorded automatically by the instrument. Impact strength (kJ/m^2^) was then calculated by normalizing the absorbed energy to the specimen cross-sectional area at the impact location.

#### 2.2.4. Statistical Analysis for Mechanical Tests

All mechanical test results are expressed as mean ± standard deviation, based on five replicates for each material variant. Statistical analyses were performed to determine the significance of differences among the composite groups. A one-way analysis of variance (ANOVA) was applied separately to the tensile properties (tensile strength, strength at break, and Young’s modulus), flexural properties (flexural strength and flexural modulus), and Charpy impact strength. The ANOVA was conducted at a 95% confidence level, and significance was determined by comparing the calculated F-ratio with the critical F-value, with corresponding *p*-values reported. In these analyses, the standard statistical parameters were evaluated, where *SS*dF, and MS represent the sum of squares, degrees of freedom, and mean square, respectively, while *F* and *p* denote the F statistic and significance level. Statistical significance was assumed when *p* < 0.05. All analyses were performed using Microsoft Excel following standard statistical procedures.

### 2.3. Microstructural Analysis

Fracture surfaces of the Charpy-tested bars were examined by field-emission scanning electron microscopy (FE-SEM; Carl Zeiss Gemini 300 (Oberkochen, Germany)). Prior to imaging, specimens were sputter-coated with a thin Au–Pd layer. Images were acquired using a secondary-electron detector at an accelerating voltage of 5–10 kV and a working distance of 5–10 mm. For each material variant, multiple fields of view were recorded over magnifications from 100× and 500×. Image assessment focused on fiber pull-out and interfacial debonding (jute/glass–PP interface), fiber rupture, matrix adhesion, and crack-path features to elucidate post-impact failure mechanisms.

### 2.4. Thermal Analyses

#### 2.4.1. Heat Deflection Temperature Tests

The HDT was measured according to ISO 75-2 [61], Method A, using an Instron Ceast HV3 HDT/Vicat tester (Turin, Italy) equipped with an oil bath. Rectangular specimens (80 × 10 × 4 mm) were tested in three-point bending mode with a support span of 64 mm. A standard load of 1.8 MPa (HDT-A) was applied, with the instrument automatically adjusting the necessary mass. The oil bath was heated at a constant rate of 2 °C/min. The HDT was recorded as the bath temperature at a deflection of 0.34 mm, in accordance with the ISO 75-2 specification. For each material condition, three specimens were tested, and the results are presented as mean ± standard deviation.

#### 2.4.2. Thermogravimetric Analysis

Thermogravimetric analysis was performed to assess the thermal stability and decomposition behavior of all composite variants. Measurements were carried out on a TA Instruments SDT 650 simultaneous DSC–TGA analyzer (New Castle, DE, USA) operated in TGA mode. Tests were conducted under flowing nitrogen, heating from 30 to 600 °C at a constant rate of 10 °C·min^−1^. Mass loss as a function of temperature and the corresponding derivative thermogravimetry (DTG, dm/dT) were recorded to identify the principal degradation steps and to determine onset/peak temperatures and residual mass at 600 °C.

#### 2.4.3. Differential Scanning Calorimetry

Thermal transitions were characterized by DSC using a TA Instruments DSC 250 (New Castle, DE, USA). Tests were performed under nitrogen purge using a standard heat–cool–heat protocol: specimens were first cooled to −50 °C, heated to 250 °C at 10 °C·min^−1^, cooled back to −50 °C at 10 °C·min^−1^, and then reheated to 250 °C at 10 °C·min^−1^. positions, and the corresponding enthalpies by baseline integration. The degree of crystallinity ($X_{C}$) of the PP phase was calculated as:

(3) $$X_{C}=\frac{∆H_{f}}{(1-w_{f})∆H_{f}^{0}}\times 100$$

where $w_{f}$ is the total fiber mass fraction (jute + glass, $w_{f}=0.2$ in all compositions), $∆H_{f}$ is the melting enthalpy value, and $∆H_{f}^{0}$ is the melting enthalpy of the 100% crystalline PP.

## 3. Results and Discussion

### 3.1. Density Measurements of the Hybrid Composites

The densities of the PP-jute-glass composites were measured according to ISO 1183-1, and the results are summarized in Table 2. A clear and systematic trend is observed as a function of the jute–glass ratio. The jute-rich formulation (J20/G0) exhibits the lowest density, while the density gradually increases with increasing glass fiber content. This behavior is consistent with the lower intrinsic density of jute compared with the polymer matrix and the glass reinforcement.

### 3.2. Mechanical Properties

#### 3.2.1. Tensile Properties

**Figure 2:**
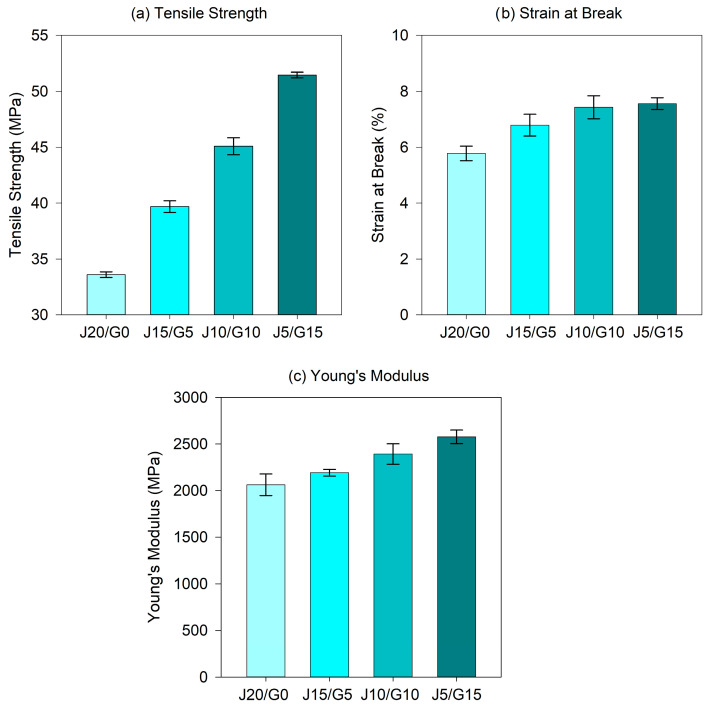
Tensile properties of PP-based jute–glass hybrid composites.

The tensile properties of the PP-based jute-glass hybrids were comprehensively assessed in terms of tensile strength, tensile strength at break, tensile strain at break and Young’s modulus, with the corresponding mean ± SD (*n* = 5) values compiled in Table 3. For clearer visualization of composition-dependent behavior across the four Jx/Gy variants, Figure 2 focuses on tensile strength, tensile strain at break and Young’s modulus, which most effectively convey the trends. The tensile strength at break is reported in the table for completeness but is not plotted because it closely tracks tensile strength for all compositions, offering little additional insight; omitting it from the figure improves visual clarity while retaining full numerical detail in the table.

The hybrid PP composite shows a statistically significant increase in tensile strength and stiffness with higher glass fiber content. This enhancement in tensile performance is primarily attributed to the superior mechanical properties of glass fibers, which possess significantly higher tensile strength and modulus than jute. The increased stiffness and strength values are indicative of more effective stress transfer from the PP matrix to the reinforcing phase, facilitated by the stronger interfacial bonding and more favorable aspect ratio distribution of glass fibers.

The tensile strength increased from 33.60 MPa for the neat jute composite (J20/G0) to 51.46 MPa for the glass-rich hybrid (J5/G15), which represents a 53.2% improvement. Similarly, Young’s Modulus showed a consistent increase, rising from 2062 MPa to 2576 MPa, equivalent to a 24.9% gain. These results demonstrate that incorporating a higher fraction of glass fiber effectively enhances the load-bearing capacity of the composites. Comparable trends have been widely reported in the literature. Sommer et al. [38] found that compression-molded PP composites exhibited significant increases in tensile strength and modulus when glass fibers replaced jute, attributing the improvement to the higher stiffness of glass fibers and better interfacial adhesion with PP. Similarly, Khan et al. [39] demonstrated that glass/PP composites possessed nearly double the tensile strength of jute/PP due to stronger fiber–matrix bonding and reduced fiber pull-out. In the present study, the tensile strength of J5/G15 exceeds 50 MPa, approaching the performance levels reported for glass-dominant PP hybrids in Khan’s work, despite the relatively low overall fiber fraction (20 wt%), which emphasizes the efficiency of the hybridization strategy adopted here. Uawongsuwan et al. [31] showed that introducing glass into long jute fiber/PP composites improved tensile modulus by 4–18% and tensile strength by up to 64%.

Notably, increasing the glass fiber content not only enhanced strength and stiffness but also did not induce tensile brittleness; instead, the strain at break rose modestly from 5.78% to 7.56%. This suggests that moderate glass incorporation sustains or even slightly improves ductility, which is favorable for semi-structural automotive applications requiring a balance between rigidity and energy absorption. Mechanistically, premature fiber rupture and interfacial debonding in jute-rich PP can trigger early damage and cap elongation, whereas the stronger glass–PP interface sustains load to higher strains before fiber failure, enabling the PP matrix to undergo additional plastic deformation. Prior studies support this behavior: limited glass additions in PP hybrids have been shown to enhance toughness or at least preserve ductility by smoothing stress distributions and mitigating early microcrack coalescence [31], while improved interfacial shear strength in glass/PP compared to jute/PP has been associated with better adhesion and delayed fiber pull-out [39]. Accordingly, the present hybrids demonstrate a favorable strength–ductility balance rather than the typical strength–brittleness trade-off.

The tensile strength values obtained for the present PP/jute–glass hybrids, ranging from 33.6 MPa for the jute-only composite (J20/G0) up to 51.5 MPa for the glass-rich formulation (J5/G15), position these materials within the mechanical performance window of widely used engineering plastics in the automotive sector. For instance, ABS typically exhibits tensile strengths of around 40–45 MPa with elongations at break between 20 and 40% [62,63]. Similarly, ASA resins generally fall in the range of 35–45 MPa tensile strength and 15–30% elongation at break [64,65]. Although the tensile strengths of the present hybrids are thus comparable to or even exceeding those of ABS and ASA, their strain at break remains considerably lower (5.8–7.6%), indicating a more brittle deformation profile relative to these commodity engineering plastics. For further context, the literature data for PP composites containing 20 wt% glass fiber (PP-GF20) typically report higher tensile strengths in the range of ≈60–80 MPa [66,67] and Young’s modulus of about 3–4 GPa [66,68]. Accordingly, the J10/G10 and J5/G15 hybrids can be positioned between the purely jute-reinforced J20/G0 composite and such PP-GF20 grades, as expected for an intermediate glass-fiber fraction.

These results highlight that hybridization with glass fiber markedly enhances the tensile performance of jute/PP composites. The J5/G15 composition reached ~51 MPa tensile strength with ~7.5% elongation at break and an increased Young’s modulus. Although this ductility is lower than that of unfilled engineering plastics such as ABS and ASA, it remains higher than the ≈1–5% elongation-at-break range commonly reported in the literature for materials that have been explicitly proposed for stiff semi-structural automotive components and interior parts [69,70,71]. Beyond strength, the inclusion of jute lowers composite density by about 5–10%, depending on the composition, enabling weight reduction and improved fuel efficiency. Thus, these bio-hybrid PP-jute-glass composites present a promising balance of strength, toughness, and sustainability, making them strong candidates for semi-structural automotive applications.

#### 3.2.2. Flexural Properties

**Figure 3:**
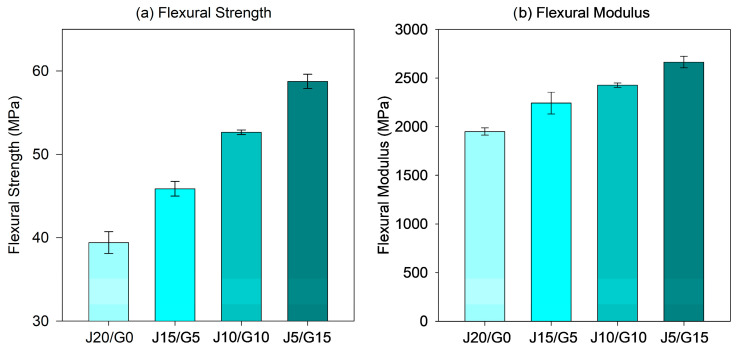
Flexural properties of PP-based jute–glass hybrid composites.

The flexural behavior of the PP-based jute–glass fiber hybrid composites is comprehensively summarized in Table 4, which reports three distinct parameters: maximum applied load, flexural strength, and flexural modulus. These values collectively capture the bending performance of the composites, with maximum applied load reflecting the absolute force sustained during testing, flexural strength quantifying the material’s resistance to bending-induced failure, and flexural modulus describing stiffness under elastic deformation. For improved clarity of the composition-dependent trends, Figure 3 highlights only the variations in flexural strength and modulus, as these two metrics most directly illustrate the strengthening and stiffening effects of glass fiber incorporation. Presenting the data in this manner provides a clearer visualization of the hybridization effect, while retaining the full set of numerical values in the table for completeness.

A statistically significant compositional dependence was observed, with flexural strength and modulus consistently increasing as the glass fiber content rose. Specifically, the maximum flexural stress increased from 39.42 MPa for the jute-only composite (J20/G0) to 58.75 MPa for the glass-rich hybrid (J5/G15), corresponding to an improvement of 49.1%. Similarly, the flexural modulus rose from 1951 MPa to 2665 MPa, reflecting a 36.6% enhancement. These findings highlight the superior stiffness and load-bearing capacity imparted by glass fibers, whose higher intrinsic modulus and improved interfacial bonding with the PP matrix facilitate more efficient stress transfer under bending.

Similar improvements have been consistently reported in the literature. Uawongsuwan et al. [31] observed that glass hybridization in long jute/PP composites increased flexural modulus by 16–30%, depending on fiber configuration. Khan et al. [41] reported flexural properties of 38 MPa and 1685 MPa for jute/PP composites containing 20 wt% fiber, which are in close agreement with the present jute-only composite (39.4 MPa and 1951 MPa at the same fiber content). Meanwhile, the glass-rich hybrid exhibited markedly higher values, highlighting once again the superior reinforcing efficiency of glass fibers over natural fibers alone. In a related study, Zhao et al. [72] investigated the flexural behavior of needle-punched jute and glass fiber mats in hybrid laminates and reported that the flexural strength of pure jute composites (37 MPa) increased by more than 60% with the incorporation of glass mats. This enhancement closely parallels the improvement observed in the present work, where the flexural strength of the jute-only composite (39.4 MPa) increased by 51% in the glass-rich hybrid (59.6 MPa). Both findings confirm that hybridization effectively combines the stiffness and load-bearing capacity of glass fibers with the sustainability advantages of jute, providing strong external validation for the trends observed in the present PP–jute–glass system. Likewise, Kshatriya et al. [43] confirmed that jute–glass PP hybrids deliver superior flexural performance compared to natural fiber-only composites, while maintaining weight reduction and sustainability benefits.

These results also indicate that the flexural performance of the glass-rich hybrid (J5/G15, 58.7 MPa strength and 2.67 GPa modulus) approaches that of conventional engineering plastics used in automotive applications. For instance, ASA typically exhibits flexural strengths in the 60–75 MPa range with a modulus around 2.2 GPa [73,74], while ABS generally falls between 45 and 65 MPa and 2.0–2.5 GPa [75,76]. Thus, the present bio-hybrid PP–jute–glass composites not only outperform the jute-only system but also achieve flexural properties comparable to ASA and ABS, positioning them as viable candidates for semi-structural automotive applications where stiffness, strength, and sustainability must be balanced.

While glass fiber addition markedly enhances the tensile and flexural properties of PP composites, the inclusion of jute provides equally critical advantages in hybrid systems. Due to its lower density, jute contributes to improved specific strength and stiffness compared to all-glass counterparts, aligning well with the lightweighting targets of the automotive sector. Moreover, as a renewable and biodegradable fiber, jute significantly reduces the environmental footprint of composites and supports life-cycle sustainability goals [7,23]. Cost competitiveness is another key benefit, since jute is considerably less expensive than glass fibers, making hybridization economically attractive. Recent studies confirm that jute–glass hybrids therefore offer not only mechanical performance improvements but also a favorable balance of specific properties, sustainability, and cost-effectiveness [34,36].

#### 3.2.3. Charpy Impact Properties

**Figure 4:**
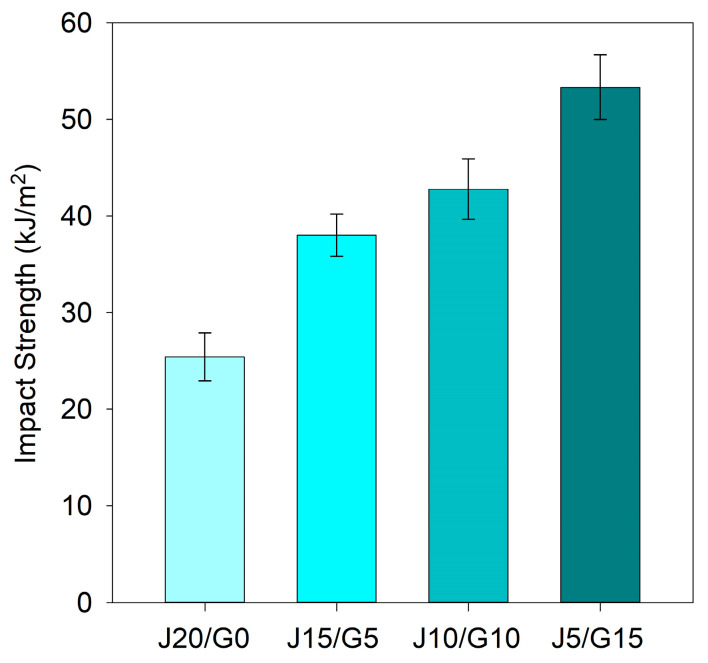
Charpy un-notched impact strength of PP-based jute–glass hybrid composites.

The unnotched Charpy impact strength results are summarized in Table 5. A statistically significant composition-dependent trend was observed: as the glass fiber fraction increased, the impact resistance of the PP-based hybrids improved significantly as seen in Figure 4. The jute-only composite (J20/G0) exhibited an impact strength of 25.41 kJ/m^2^, whereas the glass-rich hybrid (J5/G15) reached 53.34 kJ/m^2^, corresponding to an enhancement of approximately 110%. This trend confirms that hybridization with glass fiber significantly enhances the energy absorption capacity of the composites under impact loading.

The present findings align well with prior studies. Ravishankar et al. [42] reported that in PP matrix composites with a total reinforcement of 40%, replacing the jute-only system with a hybrid of 20% jute and 20% glass resulted in an increase of about 63% in impact strength, highlighting the superior reinforcing effect of glass fibers due to their higher strength and better interfacial adhesion. Similarly, Khan et al. [39] found that in composites with 50% fiber content, the impact strength was 18 kJ/m^2^ for jute/PP and 35 kJ/m^2^ for glass/PP, highlighting the superior interfacial compatibility of glass fibers. Sommer et al. [38] also reported notable improvements in energy absorption capacity with the addition of glass fibers.

When benchmarked against common automotive engineering plastics, the impact performance of the jute–glass hybrids are highly competitive. Literature reports unnotched Charpy impact strengths for ABS in the range of 34–64 kJ/m^2^ [62,77]. Although direct data for ASA are scarce, PC/ASA alloys—which are reinforced by polycarbonate—typically deliver impact strengths between 25 and 70 kJ/m^2^ [78]. Since polycarbonate generally enhances toughness, it is reasonable to assume that pure ASA would exhibit somewhat lower impact performance within this range. Accordingly, the present jute–glass hybrids can be considered well within the performance window of both ABS and ASA-based materials.

#### 3.2.4. Results of ANOVA Analysis

A one-way ANOVA was employed to quantify the effect of fiber partitioning among four jute/glass compositions on each mechanical response. The experiment followed a balanced design with five replicates per group. For tensile properties, the composition effect is unambiguous, as seen in Table 6. Tensile strength exhibits an extremely large F-ratio, F = 1180, together with a vanishingly small *p*-value, *p* = 5.58 × 10^−19^, confirming that the observed differences among formulations are not attributable to random variation. The effect is likewise significant for tensile strain at break, F = 31.31 and *p* = 6.28 × 10^−7^, and for Young’s modulus, F = 31.71 and *p* = 5.76 × 10^−7^, demonstrating that both ductility and tensile stiffness are strongly governed by how the fixed total fiber loading is distributed between jute and glass. In each case, the observed F-ratio lies well above the corresponding critical F threshold for the test, reinforcing the conclusion that composition exerts a genuine and substantial influence on the tensile responses.

Flexural responses display the same statistical picture, as shown in Table 7. Flexural strength yields F = 431.7 with *p* = 1.63 × 10^−15^, while flexural modulus yields F = 102.9 with *p* = 1.14 × 10^−10^. These results indicate that both load-bearing capacity in bending and bending stiffness are highly sensitive to fiber partitioning, with *p*-values several orders of magnitude below 0.05.

For impact performance, the un-notched Charpy impact strength also exhibits a strong main effect of composition, as seen in Table 8. The ANOVA yields F = 83.68, which is well above the critical F threshold. The very small *p*-value (*p* = 5.4 × 10^−10^) indicates that the observed differences in energy absorption across the four compositions are predominantly systematic and driven by fiber partitioning rather than stochastic fluctuations, underscoring composition as a primary determinant of impact toughness. These results collectively confirm that fiber partitioning exerts a statistically significant influence on all measured mechanical properties.

### 3.3. SEM Observation Result

**Figure 5:**
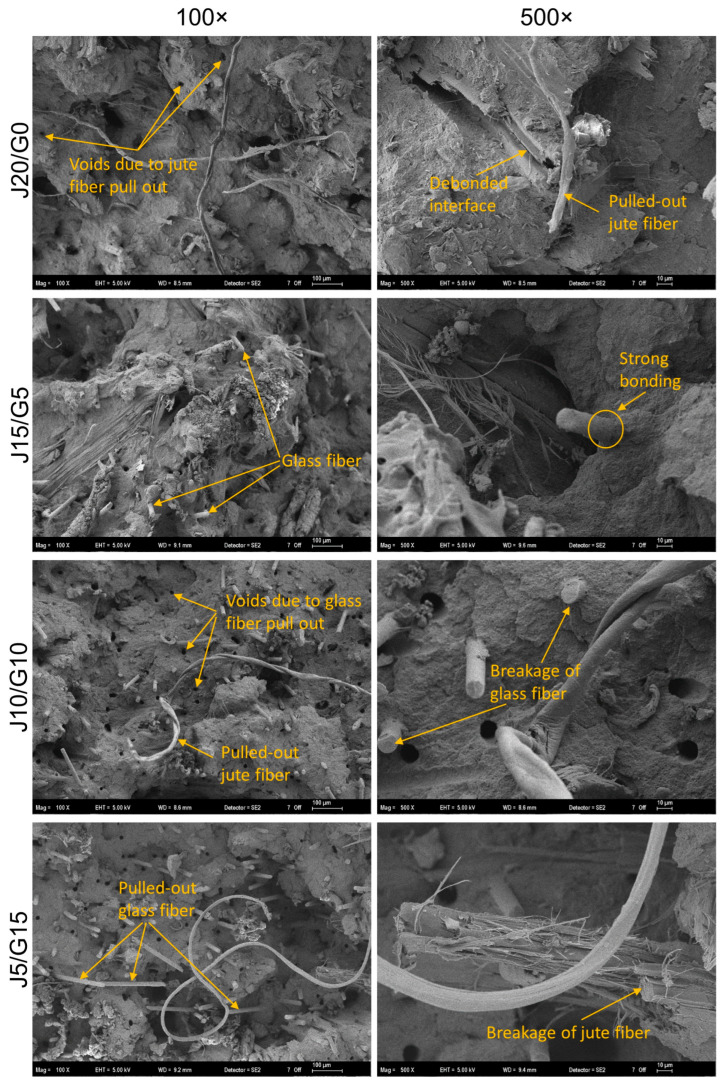
SEM images of fracture surfaces of PP-based jute–glass hybrid composites.

The fracture morphology of the composites after impact testing, as illustrated in Figure 5, reveals clear differences depending on the fiber composition. In jute-rich specimens, fracture is governed primarily by interfacial debonding and fiber pull-out. Long jute bundles withdraw from the PP matrix, leaving voids and irregular imprints at the fracture plane. The lack of resin adhering to the fiber surfaces and the abundance of empty pull-out holes reflect poor interfacial bonding, which arises from the inherent mismatch between jute’s hydrophilic nature and the hydrophobic PP matrix [79]. As a result, energy is dissipated mainly by frictional sliding of pulled-out fibers rather than by fiber rupture or cohesive matrix deformation [80].

With incremental glass addition, the fracture surface takes on a mixed-mode character (Figure 5). Jute fibers still tend to debond and pull out, leaving voids and fiber imprints, while glass fibers more frequently fracture within the matrix or remain partially embedded with resin remnants adhering to their surfaces. A similar coexistence of fiber pull-out and breakage has been widely reported in hybrid jute–glass composites, where SEM consistently shows both mechanisms active at once [81]. At the same time, several empty pull-out sites of glass fibers are also evident, showing that interfacial debonding is partially mitigated, as also reported in the literature for glass fiber–reinforced PP, where both fiber fracture and pull-out have been observed [82]. Such hybrids thus exhibit a combination of mechanisms: some regions fail by debonding and frictional sliding, whereas others show fiber rupture and improved fiber–matrix anchoring. This mixed fractography reflects a balance between energy dissipation through jute pull-out and enhanced load transfer from glass fibers, in agreement with prior reports on hybrid and glass fiber–reinforced PP composites.

As glass content dominates, fiber fracture becomes more frequent, but fiber pull-out is still readily observable (Figure 5). Numerous embedded glass cross-sections and short fiber stubs nearly flush with the matrix indicate improved load transfer, while empty pull-out holes demonstrate that debonding remains active. This dual morphology suggests a competition between fiber breakage and pull-out: stronger PP–glass bonding promotes fiber rupture, yet incomplete wetting in some regions allows fibers to disengage. Accordingly, impact energy absorption involves both frictional sliding of pulled-out fibers and cohesive matrix deformation, though with a stronger contribution from fiber fracture compared to jute-rich systems. Such mixed fracture modes—where glass fibers both break and pull out—are consistently reported in the literature [83].

Mechanistically, the progression from jute to hybrid to glass corresponds to a shift from interface-dominated failure toward fiber-dominated failure. In jute-rich systems, the hydrophilic nature of the fibers and their poor compatibility with the hydrophobic PP matrix promote debonding and pull-out, resulting in void-rich fracture surfaces. With glass reinforcement, the fracture mode increasingly involves fiber breakage and matrix-coated fiber ends, although pull-out of glass fibers is still evident, particularly when fiber length falls below the critical threshold. Hybrid composites display both mechanisms side by side, combining frictional energy dissipation from jute pull-out with the stronger anchoring capacity of glass. This progression explains the improvement in impact resistance observed with hybridization and glass addition, in line with previous reports on natural–synthetic hybrids and glass fiber–reinforced PP systems [47,79,82].

### 3.4. Thermal Properties

The thermal performance of the jute/glass fiber hybrid composites was assessed using HDT, DSC, and TGA. While thermal properties have been frequently reported for PP composites reinforced with either jute or glass fibers individually, data on their hybrid counterparts remain limited. In particular, comprehensive analyses involving these techniques for jute/glass-reinforced PP systems are scarcely reported in the literature. This section presents a comparative thermal evaluation across four hybrid formulations, with the aim of elucidating the influence of fiber composition on heat resistance, crystallization behavior, and thermal degradation characteristics, which are critical for evaluating their potential in automotive applications.

#### 3.4.1. Heat Deflection Temperature (HDT) Behavior

**Figure 6:**
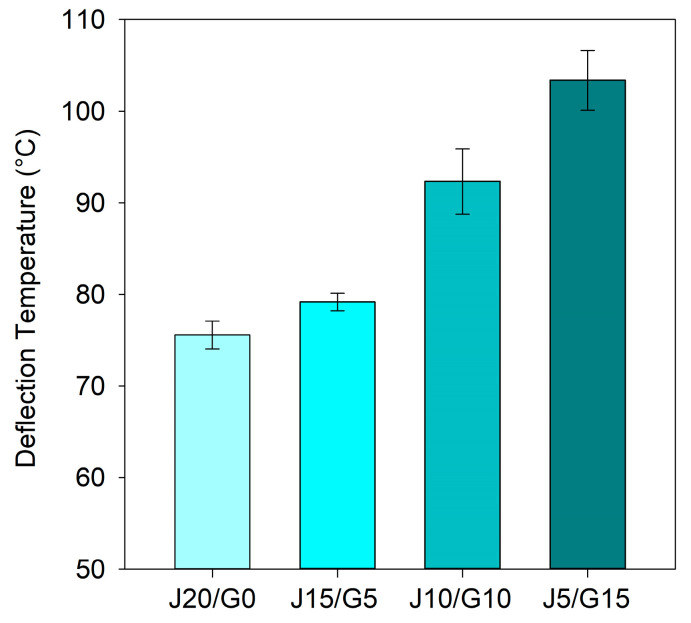
Heat deflection temperature (HDT) of PP-based jute–glass hybrid composites.

The HDT results for the hybrid specimens are shown in Figure 6, revealing a clear upward trend with increasing glass fiber content. The fully jute-based composite (J20/G0) showed the lowest HDT at 75.53 ± 1.51 °C, while gradual substitution with glass fiber led to steady improvements: 79.17 ± 0.96 °C for J15/G5 and 92.33 ± 3.56 °C for J10/G10. The highest value was obtained for J5/G15 at 103.37 ± 3.27 °C, reflecting the strong thermal stabilizing effect of glass fiber reinforcement. Notably, the standard deviations were relatively low in J20/G0 and J15/G5 but increased in J10/G10 and J5/G15. This may be attributed to the more complex internal microstructure at higher glass fiber contents, where uneven fiber distribution or localized stress concentrations could lead to greater variation in heat-induced deflection behavior.

For comparison, Nabila et al. [83] reported that 30 wt% jute fiber reinforced PP composites reached an HDT of 108.95 °C, showing the contribution of natural fibers to thermal resistance. Neat PP, on the other hand, typically exhibits HDT values around 50–60 °C [78,83]. Although HDT behavior of PP composites with single-fiber reinforcement (either jute or glass) has been reported, no prior data exist for their hybrid use. This underscores the originality of the present findings, which demonstrate that glass–jute hybridization offers a practical pathway to significantly increase HDT while balancing mechanical and environmental performance.

In this context, it is noteworthy that the HDT-A values of commonly used engineering thermoplastics such as ASA and ABS typically range between 75 and 96 °C and 80–120 °C, respectively [78]. Among the composites investigated, the glass-rich hybrid (J5/G15) clearly approached this performance range. However, even the balanced hybrid with equal jute and glass fiber content (J10/G10) exhibited comparable thermal resistance, highlighting its potential as a more sustainable alternative. These results indicate that jute/glass fiber-reinforced PP hybrids offer a practical compromise between thermal performance and environmental benefits, making them promising candidates for semi-structural applications in sectors such as automotive.

#### 3.4.2. Thermal Stability and Degradation Behavior

**Figure 7:**
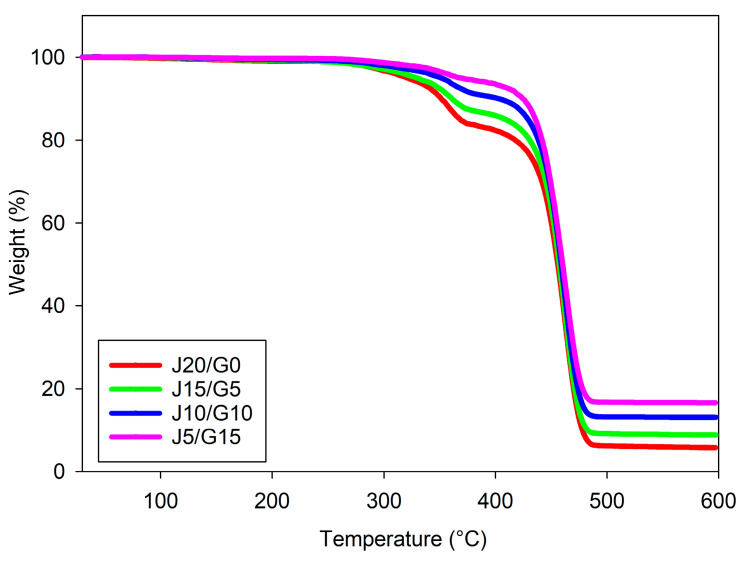
TGA curves of PP-based jute–glass hybrid composites.

TGA curves of the PP-based jute-glass hybrid composites of percent weight loss as a function of temperature are shown in Figure 7. All samples exhibited stable thermal behavior up to approximately 100 °C, after which the main decomposition phase occurred between 350 and 500 °C. The final residual mass increased in proportion to the glass fiber content, indicating the contribution of thermally stable inorganic material to the composite.

The fully jute-reinforced composite (J20/G0) displayed the lowest thermal stability among all samples. Its major degradation began around 360 °C and continued up to 500 °C, leaving a residual weight of approximately 8–9%. This behavior is consistent with the typical thermal decomposition characteristics of lignocellulosic fibers, which degrade predominantly in the 240–360 °C range due to their hemicellulose, cellulose, and lignin content [84]. The low char residue confirms the complete decomposition of the jute fibers along with the PP matrix.

As the proportion of glass fiber increased and the jute fraction decreased, an apparent improvement in thermal stability was observed. The composite with 15 wt% glass fiber (J5/G15) exhibited the highest onset degradation temperature (~400 °C) and the highest residual mass (14–15%). This trend is consistent with the non-combustible and thermally inert nature of glass fibers, together with the reduced amount of lignocellulosic jute at higher glass contents. Similar trends have been reported in the literature, where glass reinforcement has been associated with improved thermal resistance of PP composites by in-creasing char yield and delaying thermal degradation [85,86]. In particular, Jarukumjorn and Suppakarn [87] showed that, at constant total fiber loading in sisal/PP hybrids, increasing the glass fiber fraction resulted in noticeably im-proved thermal stability of the PP composites.

The hybrid composites containing both natural and synthetic fibers have been widely reported to combine the ecological benefits of lignocellulosic reinforcements with the superior thermal and mechanical stability of glass fibers. Previous studies showed that jute–glass epoxy hybrids exhibited reduced mass loss and higher residual char compared to jute-only systems, confirming the role of glass in delaying decomposition [48]. Likewise, investigations on Poly(butylene succinate) (PBS)-based jute/glass laminates revealed improved thermal stability, particularly when glass fibers were placed in outer layers, which also minimized moisture uptake and enhanced heat resistance [88]. These findings further support that hybridization with synthetic glass fibers is an effective route to overcome the inherent thermal limitations of natural fiber composites. Taken together, these findings indicate that interpreting the improved high-temperature behavior of jute/glass PP hybrids as a combined effect of (i) the lower fraction of early-degrading natural fiber and (ii) the presence of a thermally inert glass phase is consistent with the broader literature on hybrid PP composites.

These findings underline the importance of composite design tailored to application-specific thermal requirements. For instance, the glass-rich hybrid (J5/G15) may be suitable for heat-exposed automotive components such as under-hood parts or thermally stressed interior structures. Conversely, the jute-only composite (J20/G0) is more appropriate for low-temperature applications where biodegradability and resource renewability are prioritized. Meanwhile, hybrid systems like J10/G10 or J15/G5 offer a practical compromise between thermal reliability and ecological performance, making them promising candidates for lightweight and sustainable components in electric vehicles or interior paneling.

In comparison to conventional automotive plastics, widely used ABS also exhibits decomposition in a similar temperature range, with pure ABS showing a peak degradation temperature around 420 °C and leaving only ~1–2% char residue [62]. When reinforced with inorganic fillers such as nanozirconia or Polytetrafluoroethylene (PTFE), its thermal stability can be shifted up to 450–650 °C with increased char yields [76]. ASA, another automotive thermoplastic, degrades around 386 °C and fully decomposes by ~450 °C, though Poly(ether ether ketone) (PEEK) fillers can slightly enhance its stability [65]. These results suggest that the jute–glass hybrid PP composites investigated here display competitive thermal resistance relative to neat ABS and ASA, especially in terms of char residue, and thus may be considered as potential sustainable alternatives in applications where these engineering plastics are conventionally used.

#### 3.4.3. Thermal Transitions and Crystallinity Behavior

**Figure 8:**
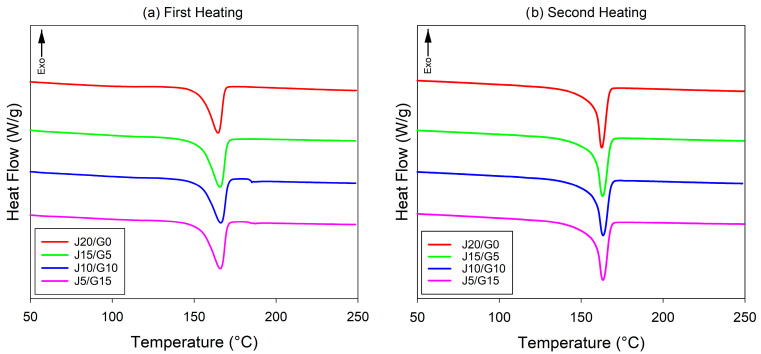
DSC curves of PP-based jute–glass hybrid composites.

DSC was employed to characterize the melting and crystallization behavior of PP-based composites reinforced with jute and glass fibers. In the present study, both the first and second heating scans were evaluated in order to capture the as-molded crystallization state of the PP matrix and the re-crystallized reference state after erasing the prior thermal history. The corresponding thermal parameters are summarized in Table 9 and depicted in Figure 8. As shown in Table 9, the crystallization temperature ($T_{c}$) and melting temperatures exhibit only minor variations with changing jute/glass ratio in both heating cycles, indicating that fiber partitioning at a fixed total loading does not strongly alter the basic crystallization kinetics of the PP matrix. This behavior is consistent with previous reports on glass-fiber-reinforced PP composites, where $T_{c}$ showed only slight upward shifts and $T_{m}$ remained nearly constant with increasing glass content [89].

The DSC first-heating results, which represent the as-molded crystallization state of the PP matrix, showed that the crystallinity ($X_{C_{1}}$) slightly increased with the incorporation of glass fiber (from 30.8% to 35.3%). The first-heating crystallinity values did not follow a strictly monotonic trend with increasing glass content or decreasing jute content, a behavior consistent with previous reports showing that nucleation and mobility-restriction mechanisms may compete and lead to non-linear crystallinity-filler content relationships [90,91]. Despite this non-monotonicity, the modest tendency toward higher $X_{C_{1}}$ in the glass-rich formulations is still expected to contribute to the observed improvements in stiffness and tensile strength. However, mechanical performance was primarily governed by the superior modulus and load-transfer capability of the glass fibers, with crystallinity acting as a secondary reinforcing mechanism.

In the second heating cycle, which reflects the re-crystallized state of the PP matrix after eliminating all processing-induced thermal history, the crystallinity values ($X_{C_{2}}$) fell within a slightly narrower and consistently higher range (≈35.6–37.6%) compared with the first-heating results. This behavior is expected for semi-crystalline polymers such as PP, because the controlled heating–cooling–reheating sequence erases the previous thermal history and allows the crystalline morphology to reorganize more uniformly compared with the heterogeneous cooling conditions experienced during injection molding [92,93]. In addition, as shown in Figure 8b, the melting temperature in the second ($T_{m_{2}}$) also exhibited only minor variations across formulations. These observations accord with Wang et al. [89], who reported that in PP composites with 10–30 wt% glass fiber, the crystallization temperature showed only a slight upward shift (115.0–116.9 °C) while the melting temperature remained nearly constant (~165 °C). Regarding the degree of crystallinity ($X_{C}$), all formulations in the present work fell within a narrow range (≈28.5–30.0%), suggesting that altering the jute–glass balance at constant total fiber loading does not substantially change the crystalline fraction of the PP matrix; the marginal increase at the highest glass content (J5/G15) may reflect a weak heterogeneous-nucleation contribution of glass fibers.

### 3.5. Application on a Prototype Automotive Part

**Figure 9:**
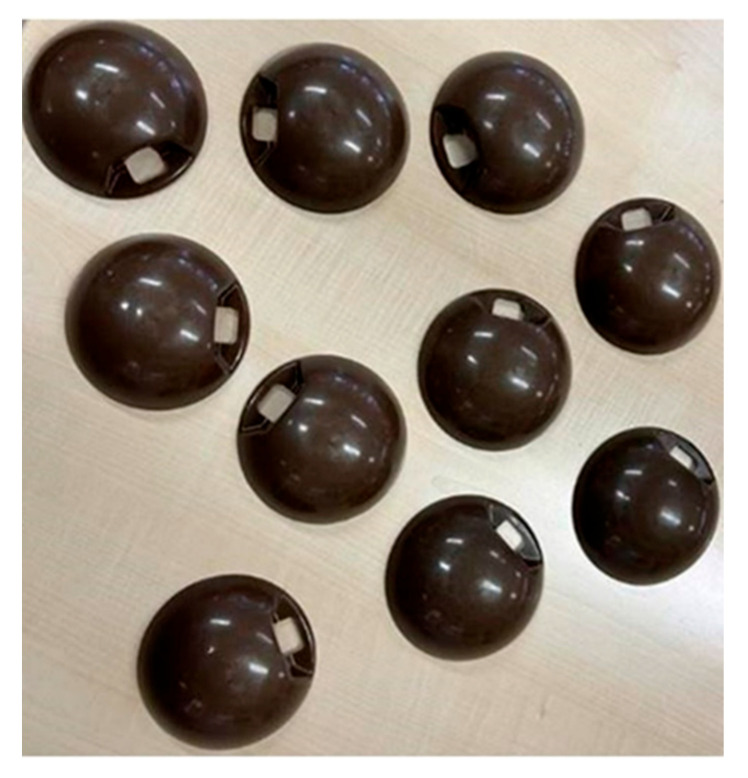
Prototype mirror cap molded from J20/G0 composite.

To further demonstrate the industrial applicability of the investigated biocomposites, the fully jute-reinforced variant (J20/G0) was successfully processed by injection molding into a prototype exterior mirror cap for a lightweight urban electric vehicle, as shown in Figure 9. During the injection molding process, no major issues were observed in terms of melt flow, cavity filling, or surface finish, indicating that the material can be reliably processed into thin-walled automotive parts. The molded mirror caps exhibited satisfactory dimensional accuracy and esthetically acceptable surface quality, confirming that the biocomposite is not only suitable at the laboratory scale but also adaptable to industrial production environments. These results demonstrate that the J20/G0 variant achieves stable processability, delivering functional and visually consistent surfaces throughout the molding cycle.

## 4. Conclusions

In this work, PP-based biocomposites reinforced with systematically varied ratios of jute and glass fibers were prepared and subjected to an extensive suite of mechanical, thermal, and morphological characterizations. The findings consistently demonstrate that the partitioning of reinforcement between natural and synthetic fibers is the principal determinant of composite performance, influencing not only tensile, flexural, and impact responses but also heat deflection behavior, thermal stability, crystallization behavior, and fracture morphology.

Mechanical testing revealed that the substitution of jute by glass fibers led to progressive and statistically significant enhancements in tensile strength (up to 53%), flexural strength (49%), and impact resistance (110%), without compromising ductility. This superior performance stems from the higher intrinsic stiffness and interfacial bonding of glass fibers, while jute contributes to weight reduction and energy absorption. The balanced hybrid formulations (e.g., J10/G10) achieved a favorable synergy by maintaining competitive strength and stiffness while preserving partial bio-based content.

Morphological investigations corroborated these macroscopic results: jute-rich composites exhibited interfacial debonding and fiber pull-out, whereas glass-rich hybrids displayed increased fiber rupture and matrix adhesion, underscoring the complementary reinforcement roles. Thermal analyses further substantiated the positive effect of hybridization. The heat deflection temperature increased from 75 °C (jute-only) to above 103 °C (glass-rich), indicating that the hybrids approach the service-temperature range of engineering plastics such as ABS and ASA, even though they do not fully match their overall performance. Thermogravimetric results confirmed enhanced thermal stability with higher glass content, while DSC analyses indicated negligible shifts in crystallization and melting temperatures, suggesting that the jute–glass ratio at constant loading exerts minimal influence on PP crystallinity.

Importantly, benchmarking against commodity engineering plastics indicated that the tensile, flexural, and impact properties of the investigated hybrids fall within the range of, and in some cases approach, those of ABS and ASA, which is encouraging for automotive applications where these polymers are widely employed in interior and exterior components. The successful processing of the J20/G0 composition into an automotive mirror cap further validated the industrial scalability and manufacturability of these materials at the component level considered in this study.

Collectively, these results suggest that jute–glass hybrid PP composites are promising candidates and potential complementary materials to conventional engineering plastics for selected semi-structural automotive applications. By uniting the ecological advantages of natural fibers with the mechanical and thermal contributions of glass reinforcement, such hybrids can contribute to weight reduction and partial bio-based content while maintaining mechanically acceptable performance at the material scale. However, we acknowledge that achieving property levels comparable to ABS or ASA in real components may require changes in part geometry, wall thickness, and processing conditions, which could introduce additional manufacturing, cost, and energy demands, and that the multi-constituent nature of the hybrids may complicate end-of-life recycling compared with single-polymer systems. In the present work, sustainability is therefore discussed in a limited sense, focusing on mass-based natural-fiber substitution, reduced composite density, and the use of a recyclable thermoplastic matrix; a full life-cycle assessment, including specific coloring, coating, and recycling scenarios, would require a dedicated study and is beyond the scope of the present contribution. Within these boundaries, the automotive-oriented benchmarking conducted here provides an initial indication that jute–glass PP hybrids can be considered for lightweight designs where mechanical reliability and incremental sustainability gains must be balanced. Future research may focus on optimizing fiber surface treatments, compatibilizer systems, processing parameters, and end-of-life strategies to further enhance interfacial adhesion, moisture resistance, long-term durability, and recyclability, thereby refining the overall environmental and functional profile of these composites.

## Figures and Tables

**Table 1:** Specimen designation and composite composition.

Specimen ID	Jute Fiber (wt%)	Glass Fiber (wt%)	PP (wt%)
J20/G0	20	0	80
J15/G5	15	5	80
J10/G10	10	10	80
J5/G15	5	15	80

**Table 2:** Density values of PP-based jute–glass hybrid composites.

Specimen ID	Density (g/cm^3^)
J20/G0	0.931 ± 0.002
J15/G5	0.953 ± 0.002
J10/G10	0.998 ± 0.001
J5/G15	1.013 ± 0.001

**Table 3:** Tensile properties of PP-based jute–glass hybrid composites.

Specimen ID	Tensile Strength (MPa)	Strain at Break (%)	Strength at Break (MPa)	Young’s Modulus (MPa)
J20/G0	33.60 ± 0.25	5.78 ± 0.26	32.25 ± 0.44	2062 ± 116
J15/G5	39.69 ± 0.52	6.79 ± 0.39	38.37 ± 0.68	2192 ± 36
J10/G10	45.09 ± 0.76	7.43 ± 0.41	43.81 ± 0.70	2392 ± 111
J5/G15	51.46 ± 0.25	7.56 ± 0.21	50.55 ± 0.22	2576 ± 73

**Table 4:** Flexural properties under three-point bending of PP-based jute–glass hybrid composites.

Specimen ID	Maximum Load (N)	Flexural Strength	Flexural Modulus (MPa)
J20/G0	65.74 ± 2.15	39.42 ± 1.29	1951 ± 37
J15/G5	77.73 ± 1.44	45.87 ± 0.86	2244 ± 111
J10/G10	89.16 ± 0.45	52.65 ± 0.27	2428 ± 23
J5/G15	99.42 ± 1.42	58.75 ± 0.85	2665 ± 60

**Table 5:** Charpy impact strength of PP-based jute–glass hybrid composites.

Specimen ID	Impact Strength (kJ/m^2^)
J20/G0	25.41 ± 2.50
J15/G5	38.02 ± 2.17
J10/G10	42.79 ± 3.13
J5/G15	53.34 ± 3.36

**Table 6:** ANOVA results for Tensile Strength, Strain at Break and Young’s Modulus.

Tensile Strength (MPa)						
Source of Variation	SS	df	MS	*F*	*p*-value	*F*-crit
Between groups	870.9	3	290.3	1180	5.58 × 10^−19^	3.24
Within groups	3.94	16	0.25			
Total	874.8	19				
Strain at Break (%)						
Source of Variation	SS	df	MS	*F*	*p*-value	*F*-crit
Between groups	9.99	3	3.33	31.31	6.28 × 10^−7^	3.24
Within groups	1.70	16	0.11			
Total	11.69	19				
Young’s Modulus (MPa)						
Source of Variation	SS	df	MS	*F*	*p*-value	*F*-crit
Between groups	766,062	3	255,354	31.71	5.76 × 10^−7^	3.24
Within groups	128,855	16	80,524			
Total	894,918	19				

**Table 7:** ANOVA results for Flexural Strength and Flexural Modulus.

Flexural Strength (MPa)						
Source of Variation	SS	df	MS	*F*	*p*-value	*F*-crit
Between groups	1049	3	349.8	431.7	1.63 × 10^−15^	3.24
Within groups	12.97	16	0.81			
Total	1062	19				
Flexural Modulus (MPa)						
Source of Variation	SS	df	MS	*F*	*p*-value	*F*-crit
Between groups	1,363,988	3	454,663	102.9	1.14 × 10^−10^	3.24
Within groups	70,707	16	4419			
Total	1,434,695	19				

**Table 8:** ANOVA results for Impact Strength.

Impact Strength (MPa)						
Source of Variation	SS	df	MS	*F*	*p*-value	*F*-crit
Between groups	2012	3	670.5	83.68	5.4 × 10^−10^	3.24
Within groups	128.2	16	8.01			
Total	2140	19				

**Table 9:** Crystallization and melting characteristics of the PP-based jute–glass hybrid composites.

**Specimen ID**	$T_{c}$ (°C)	$∆H_{c}$ (J/g)	$T_{m_{1}}$ (°C)	$∆H_{f_{1}}$ (J/g)	$X_{C_{1}}$ (%)	$T_{m_{2}}$ (°C)	$∆H_{f_{2}}$ (J/g)	$X_{C_{2}}$ (%)
J20/G0	120.34	60.09	164.59	51.07	30.83	162.51	60.38	36.46
J15/G5	120.46	66.95	165.00	58.45	35.30	163.05	59.00	35.63
J10/G10	120.28	64.14	165.50	54.62	32.98	163.35	59.30	35.81
J5/G15	120.84	64.75	165.21	57.02	34.43	163.33	62.18	37.55

## Data Availability

The raw data supporting the conclusions of this article will be made available by the authors upon request.
